# Single amino acid utilization for bacterial categorization

**DOI:** 10.1038/s41598-020-69686-5

**Published:** 2020-07-29

**Authors:** Yi-Kai Liu, Hung-Chih Kuo, Chih-Ho Lai, Chi-Chung Chou

**Affiliations:** 1 Department of Veterinary Medicine, College of Veterinary Medicine, National Chung Hsing University, Taichung, 402 Taiwan; 2 Department of Veterinary Medicine, National Chiayi University, Chiayi, Taiwan; 3 Department of Microbiology and Immunology, Graduate Institute of Biomedical Sciences, College of Medicine, Chang Gung University, Taoyuan, Taiwan

**Keywords:** Bacteria, Bacteriology

## Abstract

Despite great advancement in genetic typing, phenotyping is still an indispensable tool for categorization of bacteria. Certain amino acids may be essential for bacterial survival, growth, pathogenicity or toxin production, which prompts the idea that the intrinsic ability to utilize single amino acid under live-or-die situation could be a basis for differentiation of bacteria species. In this study, we determined the single amino acid consumption profiles of 7 bacterial species, and demonstrated that most bacteria have species-specific pattern of amino acid consumption. We also discovered that bacterial strains from different hosts, toxigenicity, and antibiotic-resistance presented distinct preference for certain amino acids. Taken altogether, the amino acid consumption profiles showed potential to be a novel tool complementary to study not only bacterial categorization but also biochemical characteristics of the bacteria such that its phenotyping can be used to uncover strategies for nutritional, pharmaceutical, taxonomic, and evolutionary aspects of bacterial researches.

## Introduction

Bacterial identification is crucial in a wide variety of applications including disease diagnosis, food safety, and environmental monitoring. In general, two distinct methods are used for bacterial identification; genotyping and the phenotyping. Genotypic methods such as DNA sequencing, refer to discrimination of bacterial strains based on their genetic content^1^. On the other hand, phenotypic traits are based on the characteristics expressed by bacterial strains, which comprise morphological, physiological, and biochemical features. For example, Analytical Profile Index (API) and Biolog system are commonly used phenotypic methods which rely on the utilization and metabolism of simple chemical compounds^2,3^. Although genotyping has recently become mainstream for bacterial strain typing, phenotypic methods still play an important role in many cases and provide crucial insights into bacterial strains. For instance, about two-thirds of genes from bacterial to mammalian cells have been shown to possess biochemical function, but only a small fraction of these genes are known to be associated with phenotypes^4,5^. Therefore, more phenotypic information is desirable in understanding the roles of those inexplicable genes^6,7^ and to discover their resultant behaviors in relations to pathology, epidemiology, and ecology.

Amino acids (AAs) are critical substances for nitrogen and carbon metabolism in bacteria. Different bacteria may have different abilities to utilize AAs depending on their genetic and adopted differences as well as the nutritional availability from the environment, rendering significant variations in their phenotypes even when they belong to the same species. So far, bacteria utilize AAs in a species-dependent manner have been reported elsewhere. For example, *Escherichia coli* can use serine (Ser), aspartate (Asp), cysteine (Cys), glycine (Gly), glutamate (Glu), and alanine (Ala) as the sole carbon and nitrogen sources during aerobic growth, while they consume Ser, Asp, Cys, and asparagine (Asn) under anaerobic conditions^8^. All these utilizable AAs are also chemoattractants for *E. coli*. In contrast, *E. coli* try to avoid high concentrations of other AAs, such as valine (Val) and leucine (Leu), which inhibit cell growth and therefore are considered as chemorepellents^8^. Different *Pseudomonas* species utilize AAs differently; for example, most *P. aeruginosa* strains can use lysine (Lys), while *P. fluorescens*, *P. stutzeri*, and *P. alcaligenes* do not. However, all *Pseudomonas* spp. have some characteristics in common, they usually consume Ala, Glu, and Asp but not methionine (Met), Cys, and threonine (Thr)^9^. *Staphylococcus aureus* often required between 3 and 12 AAs for growth, with proline (Pro), arginine (Arg), Val, and Cys being most frequently required^10,11^. In addition, Arg, Cys, and phenylalanine (Phe) are necessary for its enterotoxin production^12^. Asp, glutamine (Gln), Gly, Pro, Ser, Ala, Arg, and Asn can be used as sole carbon and nitrogen sources by *Salmonella enterica* in germinating alfalfa exudates^13^, the utilization of these AAs may be contributed to bacterial fitness during plant colonization. Toxigenic and nontoxigenic strains of *Pasteurella multocida* can grow in a specially designed minimal medium^14^, the study concluded that Met, Cys, and Glu were strictly required for growth while Leu was not necessary but stimulated growth. The previous study of amino acid auxotrophies revealed that Arg, histidine (His), Met, tryptophan (Trp), isoleucine (Ile), Leu, and Val were essential for *Enterococcus faecalis*^15^. In addition, Glu and Gln were found to be an important hub in metabolism. Although these kinds of information above are not rare, they are mostly sporadic with different experimental conditions and many cultured with multiple AAs or concurrent nutritional supplements.

Different bacterial species require different types of AAs for growth, development of resistance to a hostile environment, or toxin production, which means certain AAs are essential for some bacteria but may not be necessary for others. These facts pointed to a possibility to employ AA utilization as a phenotypic mechanism to distinguish bacterial species, especially further down to strains, types, toxigenicity, and other characteristic properties. However, a comprehensive study of simple or essential AA utilization among various bacterial strains is still lacking. For measuring AA utilization of bacteria, a method that can accurately quantitate AAs is needed. Flow-injection analysis (FIA) with electrochemical detection using copper nanoparticle-plated screen-printed carbon electrode (Cu^n^ SPE) has been successfully demonstrated for sensitive determination of all native AAs^16,17^. Compared with tag-based methods^18^, this electrochemical detection method can directly detect AAs without any chromophore or fluorophore groups. Moreover, this method is more economical and easier than spectroscopic methods such as nuclear magnetic resonance (NMR) and Raman spectroscopy^19,20^. Thus, this Cu^n^SPE detection method can be a practical tool in the measurement of AA utilization for bacterial differentiation.

The aims of this study were to exam the hypothesis that the ability of different bacteria species to utilize single AA under minimal nutritional condition (live or die) is distinct enough to comprise a new method for novel classification/typing of bacterial species, and for study of animal tropisms in the same bacterium, differentiation of toxigenicity in the same bacterium, and differentiation of the same bacterial species with or without antimicrobial resistance. Our findings may provide pivotal phenotypic information in terms of AA dependency and may uncover strategies for nutritional, pharmaceutical, taxonomic, and evolutionary aspects of bacterial researches.

## Results

### Differential utilization preference of 20 AAs among bacterial species

**Figure 1:**
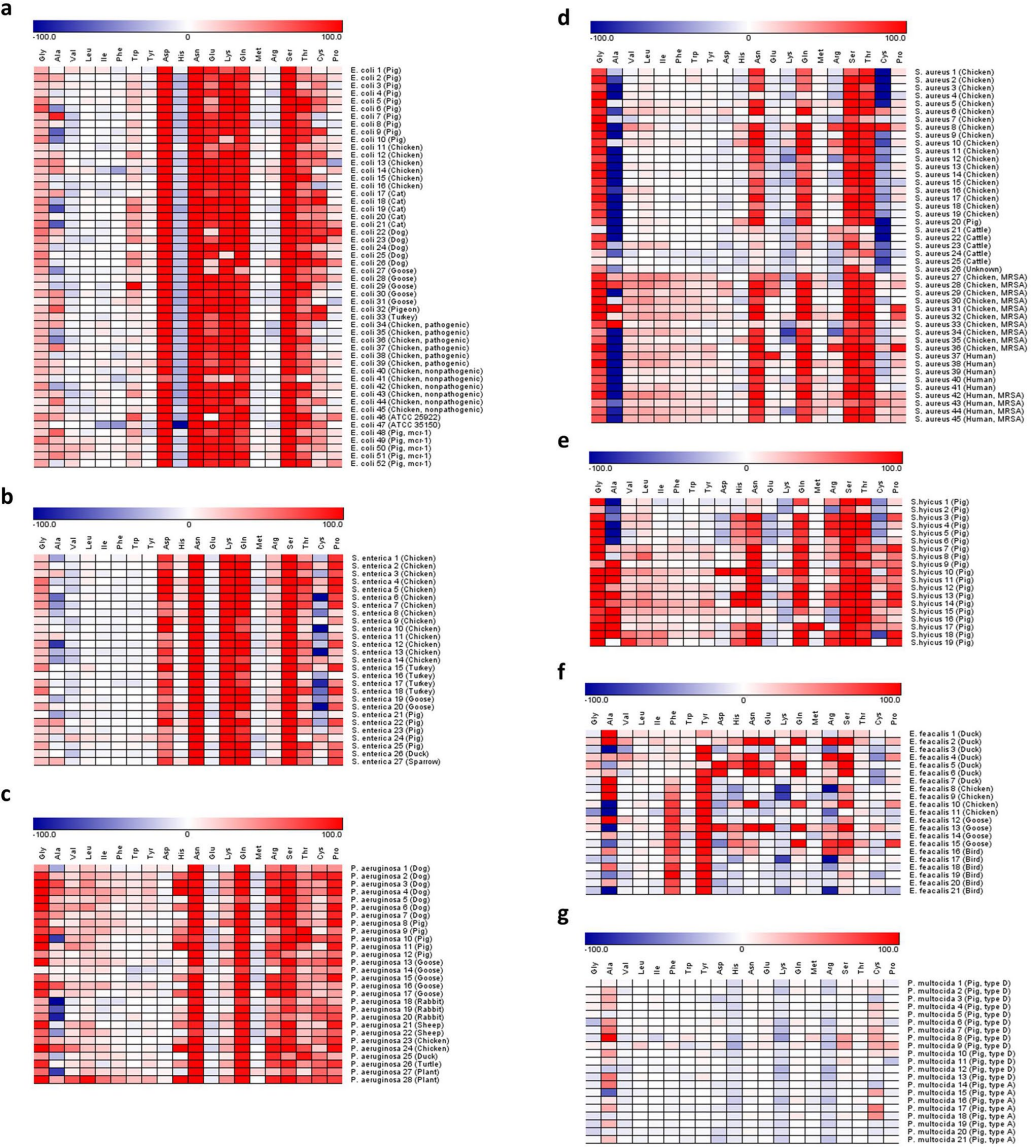
Heat map showing AA consumption profiles of 7 bacterial species. The heat map shows the consumption of 20 AAs by 213 strains from 7 bacterial species including (**a**) *E. coli*, (**b**) *S. enterica*, (**c**) *P. aeruginosa*, (**d**) *S. aureus*, (**e**) *S. hyicus*, (**f**) *E. faecalis*, and (**g**) *P. multocida* . The color gradients, which goes from white to red and white to blue, represent the level of positive consumption (0–100%) and negative consumption (0 to -100%) of AAs, respectively.

The 7 bacterial species, namely *E. coli*, *S. enterica*, *P. aeruginosa*, *S. aureus*, *S. hyicus*, *E. faecalis*, and *P. multocida*, showed distinctive consumption profiles (Fig. 1). The consumption levels of AA can be conveniently categorized into high (> 80%), intermediate (20%-80%), low (< 20%), and negative (biotransformation) (< 0%). Both *E. coli* and *S. enterica* (Fig. 1a,b) have consistently high consumption of Asp, Asn, Lys, Gln, and Ser. In addition, a high consumption of Glu and a slight negative consumption of His were observed in *E. coli*. In most *P. aeruginosa* strains (Fig. 1c), Gly, Asn, Gln, Arg, Ser, and Pro were highly consumed. *S. aureus* and *S. hyicus* (Fig. 1d,e) exhibited a similar pattern in highly consuming Gly, Asn, Gln, Ser, and Thr. The *E. faecalis* (Fig. 1f) had an inconsistent consumption pattern in comparison with other bacterial species; only Phe and tyrosine (Tyr) were found to have a relatively steady utilization. The consumption of most AAs was low in *P. multocida* (Fig. 1g); only a slight negative consumption of His, Lys, and Arg was consistently observed. Overall, nearly all strains of the 7 species have low consumption of Val, Leu, Ile, and Met.

### Differentiation of bacterial species by AA consumption profiles

**Figure 2:**
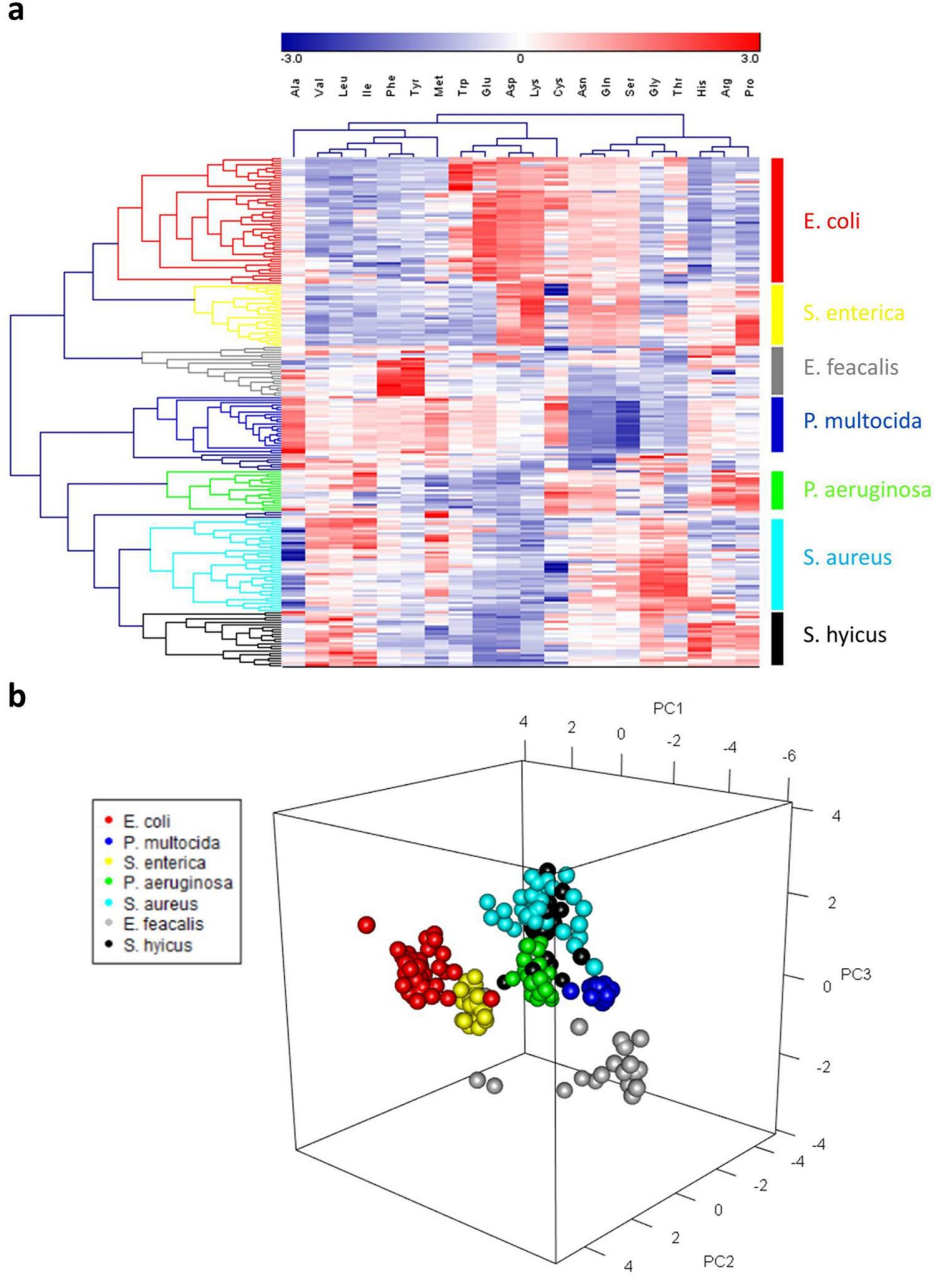
Clustering of bacterial strains by AA consumption profiles. (**a**) Hierarchical clustering and heat map of differentially consumed AAs in 213 strains. The clusters represent seven bacterial species are evident. Red represents high relative consumption and green represents low relative consumption as shown in colored scale bar above the heat map. (**b**) 3-D view of principal component analysis of 213 bacterial strains. Each spot colored according to different species indicates individual strain.

Hierarchical clustering analysis (HCA) on all 213 strains (Fig. 2a) revealed 7 clusters corresponded to the 7 bacterial species. All 52 strains of *E. coli* and all 27 strains of *S. enterica* were independently clustered with Glu being a significant factor to distinguish these two clusters. All *P. multocida* strains formed an individual cluster mainly due to the relatively low consumption of Asn, Gln, and Ser. Almost all strains of *E. faecalis* were grouped according to their specific consumption of Phe and Tyr. Most strains of *P. aeruginosa* were clustered together based on their ability to specifically consume Arg and Pro, whereas some of the strains were distributed to *S. hyicus* cluster. *S. aureus* cluster contained most of the *S. aureus* strains, but also contained some strains of *S. hyicus* and vice versa. Overall, HCA indicated that AA consumption pattern successfully categorized 100% of *E. coli* (52/52), *S. enterica* (27/27), and *P. multocida* strains (21/21), 90% of *E. faecalis* strains (19/21), 80% *S. aureus* strains (36/45), 62% of *P. aeruginosa* strains (17/28), and 52% of *S. hyicus* strains (10/19).

According to the results of HCA, 11 AAs (Gly, His, Tyr, Asp, Asn, Glu, Lys, Gln, Ser, Thr, and Pro) showing higher variance among the 7 bacterial species were selected to perform principal component analysis (PCA) for further validation to differentiate/classify bacterial species (Fig. 2b). In order to confirm whether the selected principal components (PCs) were representative enough of AA consumption data, eigenvalues and the cumulative proportion of the PCs were checked (Supplementary Fig. 1). The eigenvalues of PC1, PC2, and PC3 were all more than 1 and in combination can explain 80% of the variation, suggesting that the first 3 PCs in our PCA had an adequate reliability. The results (Fig. 2b) demonstrated that 6 species (except for *S. hyicus* which could not be readily distinguished from the *S. aureus* group) were nicely separated into 6 main groups.

### Distinction by hosts

**Figure 3:**
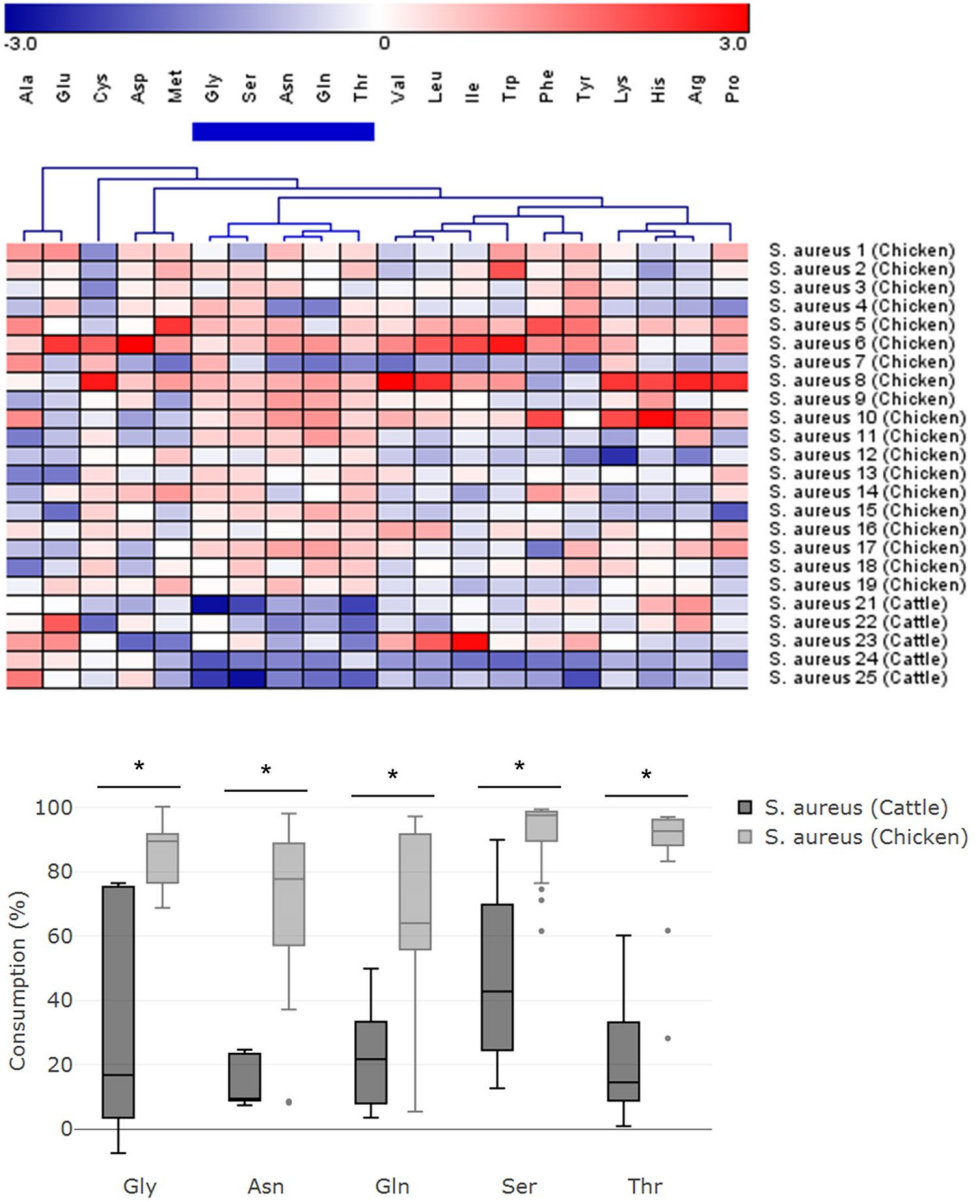
Comparative consumption of AAs among *S. aureus* strains with different host origins. The heat map shows AAs consumption by *S. aureus* strains from chicken (*S. aureus* strain 1–19) and from cattle (*S. aureus* strain 21–25). Hierarchical clustering of AAs indicated that the green cluster of cattle origin strains have a relatively lower AA consumption compared with strains from chicken, and the consumption of all five AAs in the green cluster were significantly different (*P* < 0.05) between two groups.

Consumption patterns were also analyzed based on the host origin for each bacterial species. In *S. aureus*, disparate consumption patterns were observed between the strains from chicken and cattle (both groups were not methicillin-resistant strains). The heat map and hierarchical clustering indicated that isolates from the cattle distinctively utilize Gly, Ser, Asn, Gln, and Thr lower than those from chicken with statistically significant difference (*P* < 0.05) (Fig. 3). The median [IQR] of consumption of Gly, Ser, Asn, Gln, and Thr were 17% [3 to 75], 43% [24 to 70], 9% [9 to 23], 22% [8 to 33], and 14% [9 to 33], respectively in the strains from cattle, while they were 89% [77 to 92], 98% [90 to 99], 78% [57 to 89], 64% [56 to 92], and 93% [88 to 96], respectively in the strains from chicken.

### Distinction by antibiotic-resistance

**Figure 4:**
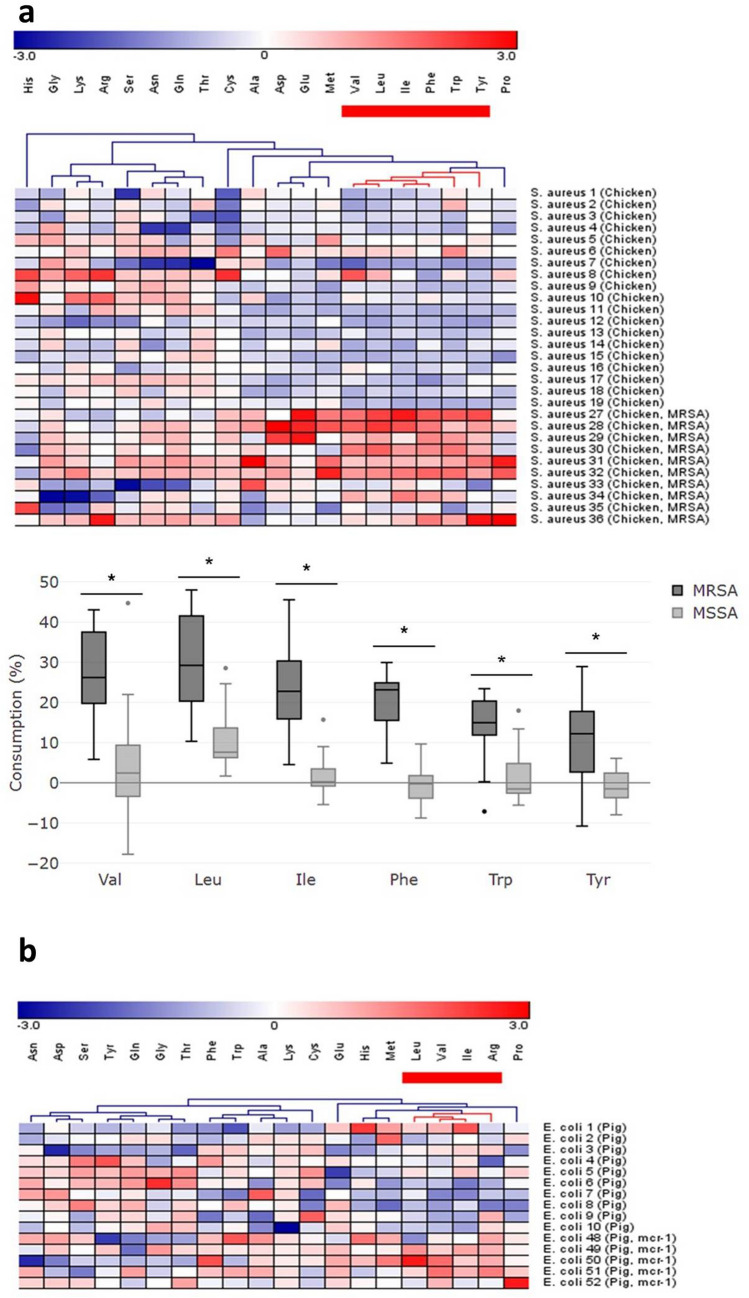
Comparative consumption of AAs among strains with and without antibiotic resistance. (**a**) The heat map shows AAs consumption by MSSA (*S. aureus* strain 1–19) and MRSA (*S. aureus* strain 27–36). Hierarchical clustering of AAs indicated that the red cluster in MRSA has a relatively higher AA consumption compared with MSSA, and the consumption of six AAs in the red cluster were significantly different (*P* < 0.05) between two groups. (**b**) The heat map of *E. coli* strains with and without *mcr*-1 gene(*E. coli* strain 1–10 and strain 48–52) and hierarchical clustering of AAs indicated that four AAs in the red cluster were consumed in different levels.

The difference in AA consumption was observed between methicillin-resistant and non-resistant *S. aureus* strains. Methicillin-resistant *S. aureus* (MRSA) utilized comparatively higher amounts of Val, Leu, Ile, Phe, Trp, and Tyr than methicillin-sensitive *S. aureus* (MSSA) (*P* < 0.05) (Fig. 4a). Median [IQR] of consumption of Val, Leu, Ile, Phe, Trp, and Tyr were 26% [20 to 38], 29% [20 to 42], 23% [16 to 30], 23% [16 to 25], 15% [12 to 20] and 12% [3 to 18], respectively in MRSA, while they were 2% [-3 to 9], 8% [6 to 14], 0% [-1 to 4], 0% [-4 to 2], -1% [-3 to 5] and -1% [-4 to 2], respectively in MSSA. Differential consumption was also observed in *E. coli* colistin-resistant (*mcr*-1 positive) strains where they utilized higher Leu, Val, Ile and Arg upon compared with non-resistant *E. coli* strains (Fig. 4b); however, only Val and Arg were significantly higher (*P* < 0.05) (Supplementary Fig. 2).

### Distinction by toxigenicity

**Figure 5:**
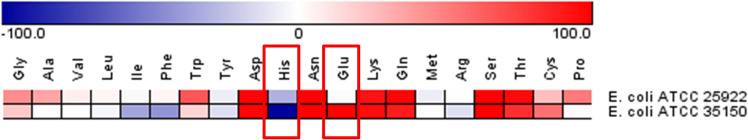
Comparative consumption of AAs between *E. coli* strains with and without toxigenicity. Consumption patterns of wild-type *E. coli* (ATCC 25922) and Shiga toxin-producing *E. coli* (ATCC 35150) are shown by heat map. These two strains have a big difference in consuming His and Glu.

The non-toxigenic strains of wild-type *E. coli* (ATCC 25922) were compared with Shiga toxin-producing *E. coli* (ATCC 35150) (Fig. 5). The His and Glu were the most distinct differences. Wild-type *E. coli* had a slight negative consumption of His and almost no consumption of Glu, whereas Shiga toxin-producing *E. coli* had almost 100% negative and positive consumption of His and Glu, respectively.

### Decision tree strategy based on specific AA utilization for bacterial differentiation

**Figure 6:**
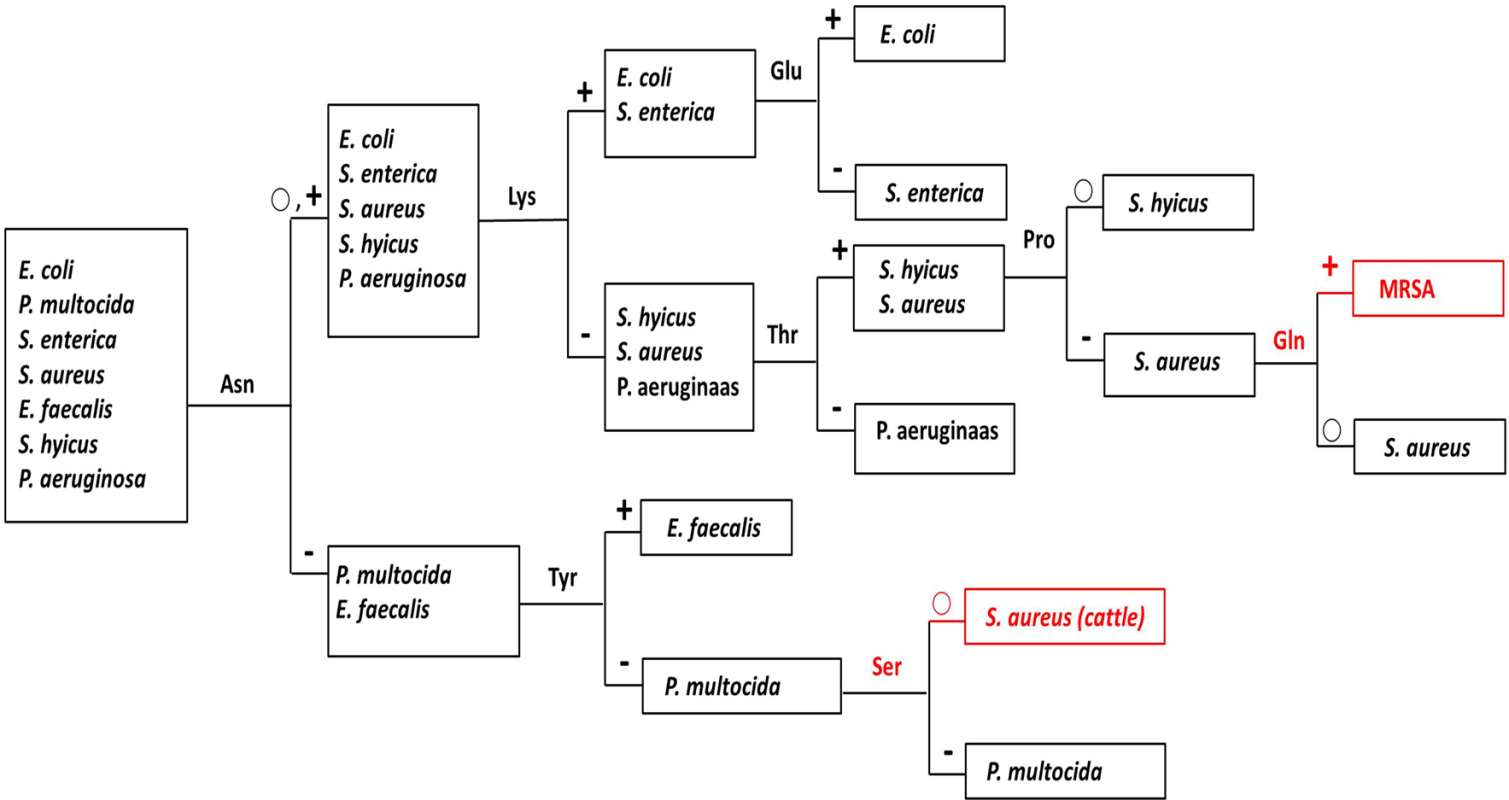
Decision tree for differentiation of bacterial species. A flow chart of the identification process based on the consumption level of 8 AAs can efficiently identify 7 bacterial species, cattle *S. aureus* and MRSA. − indicates the consumption is less than 50%; ○ indicates the consumption is from 50 to 75%; + indicates the consumption is more than 75%.

Based on the overall analysis of AA utilization, we discovered that 8 AAs, namely Asn, Lys, Tyr, Glu, Thr, Ser, Pro, and Gln can be used to make a decision tree for quick differentiation of these 7 bacteria species in a blind test situation (Fig. 6). These 7 bacterial species could be successfully differentiated in 4 serial decision making processes by using 50% or 75% as the cut-off consumption level (Table 1 and Fig. 6). For instance, an unknown bacterial strain is first tested by Asn, if the consumption is over 50%, it is further tested by Lys; and if the consumption is over 75%, then it can further be tested for Glu, if the Glu consumption is again over 75%, then this test bacterium is determined to be *E. coli,* otherwise (if the Glu consumption is less than 75%) the bacterium is deemed to be *S. enterica*. The decision tree can also differentiate MRSA versus chicken MSSA by the utilization level of Gln.

**Table 1:** Average consumption of AAs. The average AA consumption profiles of *E. coli* (n = 52), *P. multocida* (n = 21), *S. enterica* (n = 27), *P. aeruginosa* (n = 28), *S. aureus* (n = 20), cattle *S. aureus* (n = 5), MRSA (n = 10), *E. faecalis* (n = 21), and *S. hyicus* (n = 19). − indicates the average consumption is less than 50%; ○ indicates the average consumption is from 50 to 75%; + indicates the average consumption is more than 75%.

	Gly	Ala	Val	Leu	Ile	Phe	Trp	Tyr	Asp	His
*E. coli* 1–52	−	−	−	−	−	−	−	−	+	−
*P. multocida* 1–21	−	−	−	−	−	−	−	−	−	−
*S. enterica* 1–27	−	−	−	−	−	−	−	−	○	−
*P. aeruginosa* 1–28	○	−	−	−	−	−	−	−	−	−
*S. aureus* 1–20	+	−	−	−	−	−	−	−	−	−
*S. aureus* 21–25	−	−	−	−	−	−	−	−	−	−
*S. aureus* 27–36	+	−	−	−	−	−	−	−	−	−
*E. feacalis* 1–21	−	−	−	−	−	○	−	+	−	−
*S.hyicus* 1–19	+	−	−	−	−	−	−	−	−	−

	Asn	Glu	Lys	Gln	Met	Arg	Ser	Thr	Cys	Pro
*E. coli* 1–52	+	+	+	+	−	−	+	○	−	−
*P. multocida* 1–21	−	−	−	−	−	−	−	−	−	−
*S. enterica* 1–27	+	−	+	+	−	−	+	−	−	○
*P. aeruginosa* 1–28	+	−	−	+	−	○	+	−	−	○
*S. aureus* 1–20	○	−	−	○	−	−	+	+	−	−
*S. aureus* 21–25	−	−	−	−	−	−	○	−	−	−
*S. aureus* 27–36	+	−	−	+	−	−	+	+	−	−
*E. feacalis* 1–21	−	−	−	−	−	−	−	−	−	−
*S.hyicus* 1–19	○	−	−	○	−	−	+	+	−	○

## Discussion

In this study, an AAs consumption profile was established for phenotypic categorization of bacteria (Fig. 1). Different from other common bacterial phenotyping methods such as API or the Biolog system, which are based on detection of enzymatic activity related to biological transformation of carbohydrates, proteins, or organic acids, our method directly and quantitatively analyzes the consumption levels of specific AAs. The method described here can afford a more focused fingerprinting of bacterial metabolism of AAs and complement other phenotyping methods. The ease and simplicity of this method allow rapid and continuous detection of AAs without any derivatization or complex sample processing. For reliable detection, we injected 1.5 mM Gly as an internal standard before and after the detection of each AA (Supplementary Fig. 3), and set the error range of the internal standard within 15%. With this precision of quantitative analysis, the exact difference in consumption levels and thus their phenotypic properties, can be explicitly distinguished among bacterial species. However, the major drawback of our method is that the bacteria must survive and grow in liquid media (broth cultures); for those that can only grow on agar plates, gas phase analysis^21,22^ might be an alternative.

By determining each consumption level for the 20 AAs, the utilization phenotype and nutritional requirements of bacteria can be understood. Among 7 bacterial species, 5 species, namely *E. coli*, *S. enterica*, *P. aeruginosa*, *S. aureus*, and *S. hyicus*, showed consistently high consumption of 3 neutral polar AAs (Ser, Asn, and Gln). All 3 AAs play a crucial role in bacterial metabolism; for instance, Ser can be catabolized by L-serine dehydratase to produce pyruvate and ammonia, which is important for bacteria to regulate nitrogen balance and maintain extracellular pH^23,24^. Likewise, Gln and Asn are required as nitrogen donors for many important biosynthetic reactions in bacterial cells, including nucleic acid synthesis and transamidation reactions^25,26^. Previous works also demonstrated that Ser, Asn, and Gln could be rapidly utilized and metabolized by *E. coli* and other small intestinal bacteria^27,28^. All 7 species in our study showed low consumption of branched-chain amino acids (BCAAs) (Val, Leu, and Ile). Although BCAAs have been reported to play an important role in bacterial protein synthesis and maintenance of central metabolism^29–31^, studies in pig small intestine revealed that BCAAs are largely degraded by intestinal mucosal cells instead of the microbiota, which may suggest that either the bacteria could not utilize a great amount of BCAAs or a small amount of BCAAs would be sufficient to support the growth of these bacteria^32,33^. Thus, significant consumption of extracellular BCAAs could not be observed in our current study. Both Glu and Asp were largely consumed by *E. coli* in the current study; this may be related to the presence of glutamate transporters importing extracellular Glu and Asp into the cells^34^. Several L-glutamate transport systems have been identified in *E. coli*, such as a sodium-dependent transport system for Glu, a binding protein-dependent Glu/Asp transport system, and a proton symport system for Glu and Asp^35–37^. High levels of Lys were consumed only by *E. coli* and *S. enterica;* the possible reason might be that lysine decarboxylase’s activity is enhanced for tolerating the acidic environment caused by the metabolic byproducts produced from cultivating *E. coli* and *S. enterica*^38,39^. The observation that Gly was largely consumed by *S. aureus* and *S. hyicus* might be related to the formation of pentaglycine cross bridge, which is required for linking glycan chains into a two- or three-dimensional network in the biosynthesis of the peptidoglycan cell wall, and the absence of a pentaglycine cross bridge would inhibit the survival of *S. aureus* cells^40–42^. Collectively, the preferences for AAs are consistent enough such that categorization of bacteria by their intrinsic ability to utilize a simple form of nutrient becomes a feasible new way to complement genetic typing. Further validation by HCA and PCA pointed out the critical AAs with prominent effect on bacterial differentiation (Fig. 2). Most clusters of bacterial species identified by both analyses were well-separated except for the *S. hyicus* and *S. aureus*. Both bacteria belong to the genus *Staphylococcus* with high genetic similarity and biochemical properties^43^. In fact, detection of *S. hyicus* by API 20 has been reported to show low specificity^44^, thus, it is not totally unique that they shared similar AA consumption patterns. Nevertheless, a greater consumption of Arg and Pro by most *S. hyicus* strains in comparison to that by *S. aureus* strains was observed (Fig. 1d,e), which may carry biochemical significance associated with their coagulase productivity. *S. aureus* and *S. hyicus* are classified as coagulase-positive and coagulase-variable staphylococci, respectively, based on their ability to produce extracellular coagulase that can convert fibrinogen to fibrin^45^. Studies have suggested that Arg is an important alternative substrate to coagulase-negative staphylococci^46^, hence, consumption of Arg by most *S. hyicus* strains may possibly be one factor contributing to the variance in coagulase production. While this speculation requires further elucidation, our AA consumption profiles showed great potential for studying the biochemical characteristics of bacteria.

Some of the AA utilization determined in our study were not in agreement with those in previous reports. For instance, Met and Glu were regarded as necessary elements for *P. multocida* growth^14^, however, we did not observe the consumption of these AAs in our study. Similarly, a large amount of Arg was not consumed by *S. aureus*^10^. The discrepancy might be due to several reasons. First, the nutritional environment of the culture media may influence the metabolic state of the bacterial cells, resulting in different bacterial phenotypes^47^. In this study, the minimal nutritional medium (MNM) used for determining AA consumption only contained very minimal amounts of glucose and salt for energy source and for maintaining osmotic pressure, respectively. The nutritional sources in MNM are relatively poor in relation to media used in other studies, which possibly forced the change in bacterial metabolism. Second, the AA requirements are different during each growth phase: bacteria in the exponential phase need more AAs for cell growth and division than those in the stationary phase. Third, specific substances are required when bacteria utilize AAs,for example, *S. aureus* S-6 needs biotin as a growth factor when Glu is used as carbon source^48^. One interesting observation here was that some AAs presented negative consumption, which technically was due to a larger electrochemical current value of the samples in comparison to the positive control (Supplementary Fig. 3). Taking *E. coli* for example, a slight negative consumption of His was consistently observed in each strain. This phenomenon may suggest that certain AA metabolite(s) which could also produce electrochemical signals were formed upon utilization of His; further identification of metabolites is needed to confirm this deduction. It is noteworthy that the negative value was not merely a measurement of consumption but likely the sum of processes involving both the consumption and production of electroactive metabolite(s), which taken together might be better represented by the term “biotransformation.” These observations imply that the nutritional requirement of a bacterium is the result of complex evolutionary processes that cannot be deciphered or is beyond the understanding of current knowledge. Therefore, this study provided a scenario that indicates how different bacterial species react to its survival in a nutrient-scarce situation. In fact, a partial parallel study using only a single AA without MNM also showed very similar results (Supplementary Fig. 4).

Other potential applications of the current method were noticed during the study. First, *S. aureus* showed distinctive consumption profiles between different hosts, i.e., cattle and chicken (Fig. 3). Almost no AAs were consumed to a significant degree by *S. aureus* isolated from mastitis cow milk. Although a previous study suggested that strains from milk required several AAs for growth, milk is normally deficient in free AAs^49^ so alternative sources of nitrogen might be available for sustained growth of these isolates. Next, a relatively higher level of BCAA consumption was observed in MRSA and colistin-resistant *E. coli* compared to that in non-resistant strains (Fig. 4). Antibiotic-resistant strains were suggested to have reduced metabolism due to defective central metabolic pathways of glucose or AA (e.g. Ala)^50,51^. It is feasible that BCAAs, which also have a significant role in maintaining central metabolism, showed higher utilization by antibiotic-resistant strains. Interestingly, we found that human *S. aureus* and chicken MRSA strains had similar specific AAs consumption (Fig. 1d; whether the similarity of AA consumption among hosts represents a higher possibility of disease spreading (cross-host movement remains to be elucidated, and is certainly worth further study. Finally, most of the *E.coli* isolates in this study consumed a high level of Glu and a prominent difference in the consumption of His and Glu was found between the Shiga toxin-producing and wild-type *E. coli* (Fig. 5). *E. coli* mutant strains have been reported to take up Glu at a much faster rate than wild-type strains, which can be attributed to changes in cell permeability^52^. The glutamate decarboxylase (GAD) system was also reported to play an important role in the resistance to multiple environmental stresses^53^. Collectively, through these preliminary observations, we believe that the AA consumption profile holds reasonably good potential as a tool, complementary to other bacterial typing methods, for studying specific bacterial characteristics/traits. A larger-scale investigation is warranted to strengthen the power of the above claims.

Based on the consumption profiles, we were able to create a simple and straightforward strategy for differentiation of bacterial species and/or strains with just a few AAs (Fig. 6). The prerequisite is to decide on the adequate consumption level for differentiation: for instance, 50% or 70% of consumption as the cut-off points. Although the efficacy could be improved by larger sample numbers, this prospective strategy should benefit bacterial identification and research on bacterial metabolism.

In conclusion, we applied FIA-ED to quantitatively determine single AA consumption profiles of bacteria and revealed their characteristic preferences in consuming AAs. AA phenotyping might be a potential way to differentiate bacterial species and with larger-scale investigations, might also reveal a feasible way to study/categorize the development of host tropisms, toxin production, and drug resistance. Additionally, this AA phenotyping determined not only the nutritional requirements but also in part the metabolism of AAs by bacteria (such as in the negative consumption). Therefore, bacterial strains may be categorized as “specific AAs utilizing strains” or “non-utilizable strains,” which places emphasis on both the nutritional and/or metabolic phenotyping regardless of their original species. Once the relationships between AA utilization and its characteristic roles in pathogenesis, toxogenesis, or resistance can be established, this categorization will be a more powerful and informative way to describe a bacterium. Expanding on the current study will offer more phenotypic information, which can afford researchers a new perspective in analyzing microbiological data derived from biochemical and molecular biological techniques. Specific relationships acquired from this kind of study could shed more light on bacterial metabolism, bacterial adaptation to host, and development of toxigenicity and antibiotic-resistance. They may also help develop novel strategies for treating infections. For example, offering specific AA(s) that are highly or differentially utilized by the resistant strains may alter their bacterial metabolism and thereby their antibiotic susceptibility^51^. Furthermore, it might be possible to develop media formulation to selectively inhibit or enhance the growth of specific bacterial strains for targeted studies.

## Methods

### Bacterial strains

A total of 213 strains in 7 bacterial species and different hosts were collected for this study. All strains were identified to gene level by 16S rRNA gene sequencing. Bacterial species include *Escherichia coli* (52 strains), *Pasteurella multocida* (21 strains), *Salmonella enterica* (27 strains), *Pseudomonas aeruginosa* (28 strains), *Staphylococcus aureus* (45 strains), *Enterococcus faecalis* (21 strains), and *Staphylococcus hyicus* (19 strains). All clinical isolates were isolated from diseased animals in outbreak farms or veterinary hospitals, and the standard strains (ATCC 25922 and ATCC 35150) were purchased from American Type Culture Collection (Manassas). The information of all strains used in this work was listed in Supplementary Table 1.

### Preparation of amino acids (AAs)

All AAs except for Gly were purchased from Applichem GmbH (Darmstadt, Germany). Gly was purchased from GERBU Biotechnik GmbH (Heidelberg, Germany). All AAs were dissolved in MNM to a final concentration of 3 mM (Tyr was first dissolved at 55 °C). MNM was prepared by adding 0.243 g K_2_HPO_4_, 0.234 g C_6_H_12_O_6_ and 0.491 g NaCl to 1,000 mL of deionized water (1.4 mM K_2_HPO_4_, 1.4 mM C_6_H_12_O_6_ and 8.4 mM NaCl), and then filtered by 0.22 μm syringe filters with nylon membrane (Pall Corp., USA).

### Culture medium and growth condition

All bacterial strains were first recovered from its freeze-dried or frozen condition, and inoculated in Tryptic soy agar (TSA) for *S. enterica*, *S. aureus*, *S. hyicus*, *E. faecalis* and *P. aeruginosa*, in Luria agar (LA) for *E. coli*, and in Todd Hewitt agar for *P. multocida*, and incubated at 37 °C overnight (18 h). On the next day, a single colony was picked and inoculated in 5 mL of Tryptic soy broth (TSB) for *S. enterica*, *S. aureus*, *S. hyicus*, *E. faecalis* and *P. aeruginosa*, in Luria broth (LB) for *E. coli*, and in Brain heart infusion (BHI) broth for *P. multocida*, and incubated at 37 °C under shaking (180 rpm) for 18 ± 1 h. After that, 400 μL of the cultured broth was subcultured in 10 mL of respective broth and incubated at 37 °C under shaking (180 rpm). Bacterial cells were harvested by centrifugation (3,500 rpm, 10 min) when OD_600_ reaches 0.8. The pellet was washed by 5 mL of the MNM twice for removing the original broth that would interfere with the electrochemical signal (see below). The washed pellet was subsequently grown in tube each containing 3 mM of single AA solution. The bacterial suspension was adjusted to 1 × 10@@@9 CFU/mL and then incubated at 37 °C, 180 rpm for 18 h. After growth, the bacterial cultures were centrifuged (3,500 rpm, 10 min) and the supernatant was used for AA analysis.

### Electrochemical analysis of amino acids

Flow-injection analysis (FIA) with electrochemical detection (FIA-ED) was used to conduct chronoamperometric experiments (*i-t*) and quantification of AA as previously described^17^. In brief, the FIA system consists of a Hitachi L-6200 intelligent pump drive, a Shimadzu SIL-10A Auto Injector with an interconnecting Teflon tube and a specially designed electrochemical flow cell (Zensor R&D, Taichung, Taiwan) incorporating a three-electrode system consisting of a working electrode (Cu^n^-SPE, copper nanoparticle-plated screen-printed electrode, geometric area of 0.2 cm@@@2), an Ag/AgCl reference electrode, and a platinum auxiliary electrode (geometric area of 0.07 cm@@@2). The detection condition using FIA system were: mobile phase: 0.01 M, pH 7 phosphate buffered saline (PBS), analytical potential: 0.15 V vs. Ag/AgCl, scan rate: 0.05 V/s, flow rate 0.5 mL/min, injection volume 20 μL. The internal standard is 1.5 mM Gly. Chronoamperometric signals for quantification were analyzed by CHI1021C electrochemical analyzer with CHI system software (CH Instruments, Austin, TX, USA). For determination of AA utilization, the percentage of AA consumption (A%) were determined by the equation

$$A\;\left( \% \right) = 100 - \left( {\frac{T - N}{P} \times 100} \right)$$

where N is the electrochemical current value of the negative control, in which bacterial strain was grown in the medium containing only MNM; P is the current value of the positive control, which contained 3 mM of each AA solution without bacterial inoculation; T is the average current value of replicate samples containing both bacteria and AA solution.

### Statistical analysis

Genesis software version 1.8.1 was used to create heat maps and HCA^54^. In HCA, each AA consumption data was first standardized by z-score normalization and then used to perform average linkage distance hierarchical clustering. PCA was performed using the prcomp function in R version 3.5.3 to investigate similarities between individual samples based on correlation^55^. The rgl package was used to create interactive three-dimensional visualization of data. To assess the difference between groups, SPSS software (v.25) was used to perform the Mann–Whitney U test for non-normally distributed data. The data is represented as the median with interquartile range (IQR), and box plots were created for visualization of the data using R.

## Supplementary information

Supplementary file1
