# Supplementary material for: Single amino acid utilization for bacterial categorization
Source: Sci Rep. 2020 Jul 29;10:12686. doi: 10.1038/s41598-020-69686-5 (PMC7391690; doi:10.1038/s41598-020-69686-5)
Supplement: Supplementary file 1 — Supplementary file1 [file 41598_2020_69686_MOESM1_ESM.pdf]

Supplementary Information:

Single amino acid utilization for bacterial categorization

Yi-Kai Liu, Hung-Chih Kuo, Chih-Ho Lai, Chi-Chung Chou

(a)

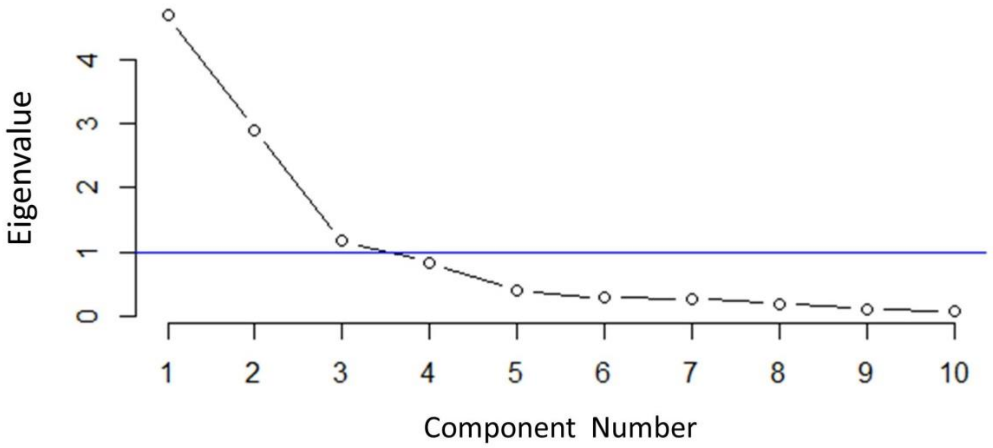

(b)

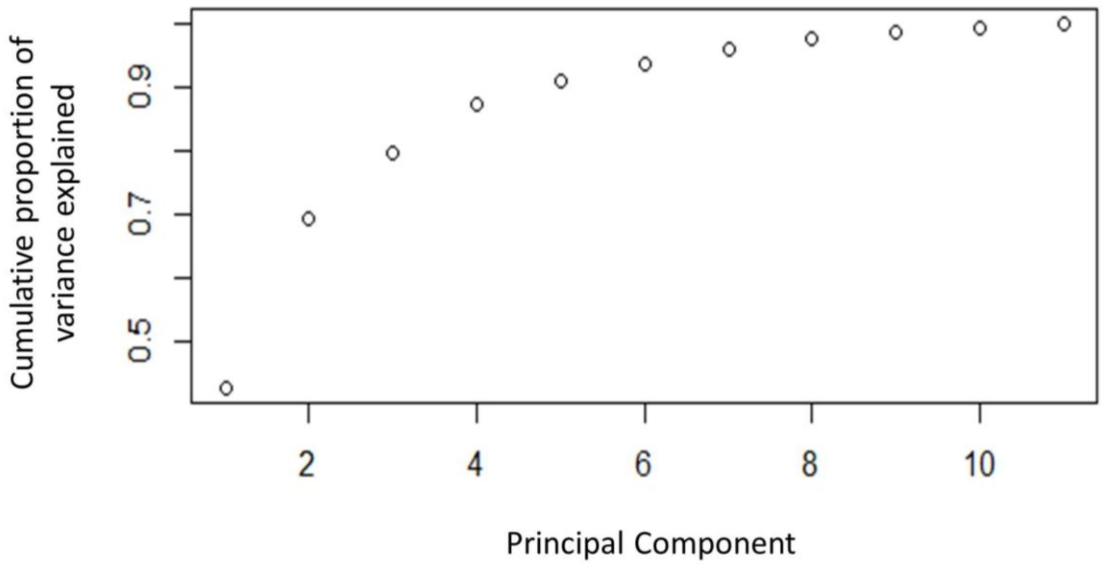

Supplementary Figure 1. Evaluation of PCA. (a) The scree plot graphs the eigenvalue against the component number. A component that displays an eigenvalue greater than 1 accounts for a greater amount of variance than had been contributed by one variable, and therefore accounts for a meaningful amount of variance. (b) Cumulative proportion of variance explained with PCA. Together the first three components (PC1-3) account for 80% of variance.

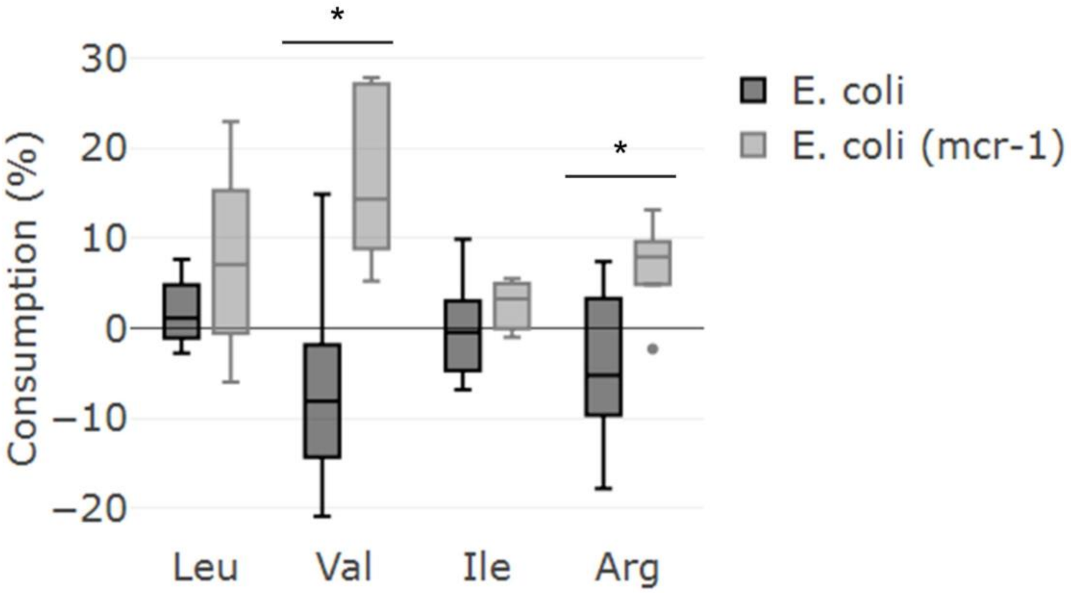

Supplementary Figure 2. Comparative consumption of AAs between *E. coli* strains with and without *mcr*-1 gene. Median [IQR] of consumption of Leu, Val, Ile, and Arg were 1% [-1 to 5], -8% [-14 to -2], 0% [-5 to 3], and -5% [-10 to 3] respectively in strains without *mcr*-1 gene, while they were 7% [-1 to 15], 14% [9 to 27], 3% [0 to 5], and 8% [5 to 10] respectively in strains with *mcr*-1 gene. The consumption of Val and Arg were significantly different ($P < 0.05$) between two groups.

(a)

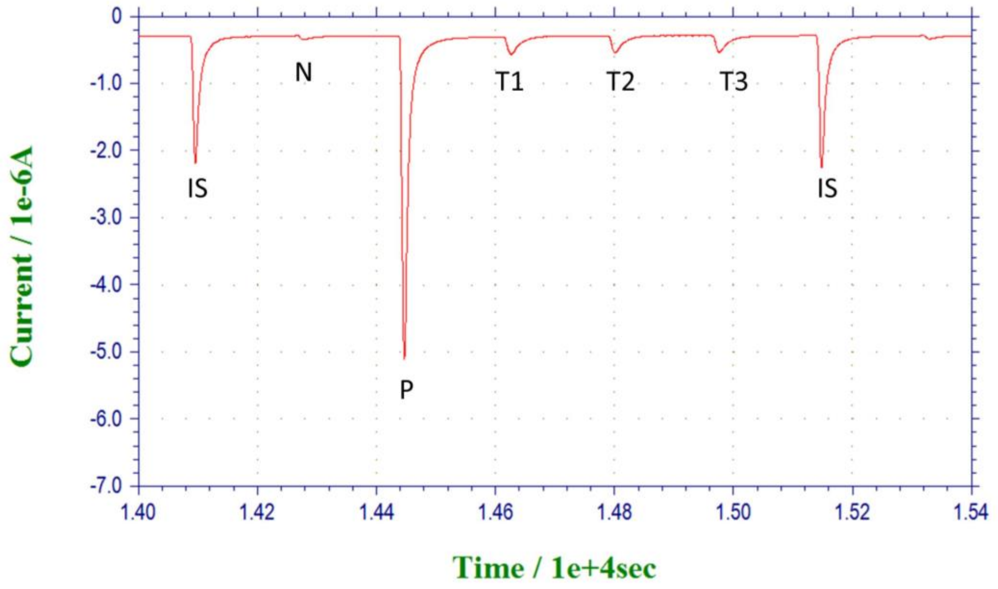

(b)

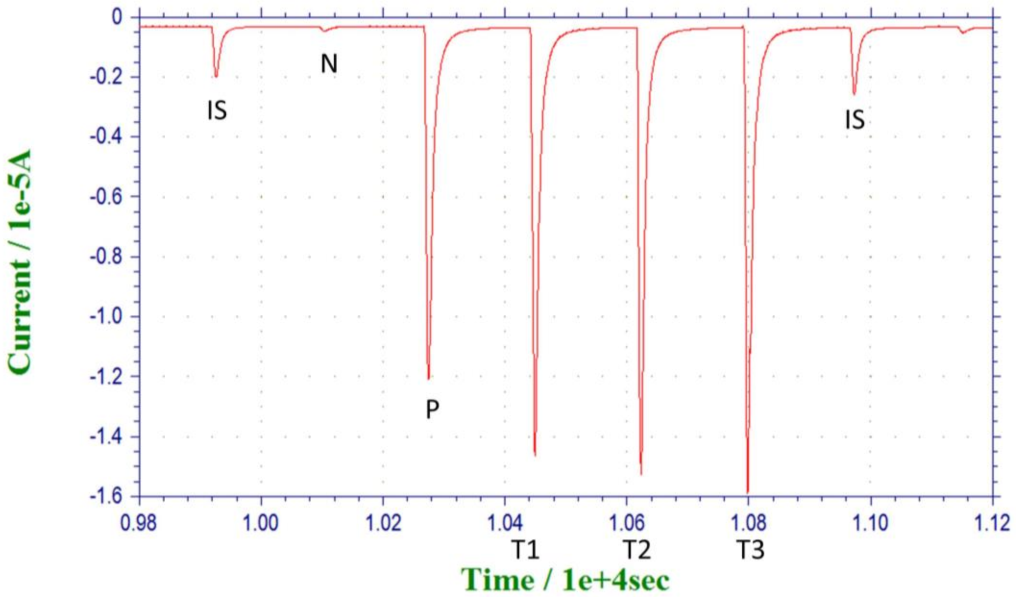

Supplementary Figure 3. Chronoamperometric signals in amino acid detection. IS, N, and P are the electrochemical current value of the internal standard, the negative control, and positive control respectively. T1, T2, and T3 are the current values of triplicated samples. (a) The detection of high consumption of AA. (b) The detection of negative consumption of AA.

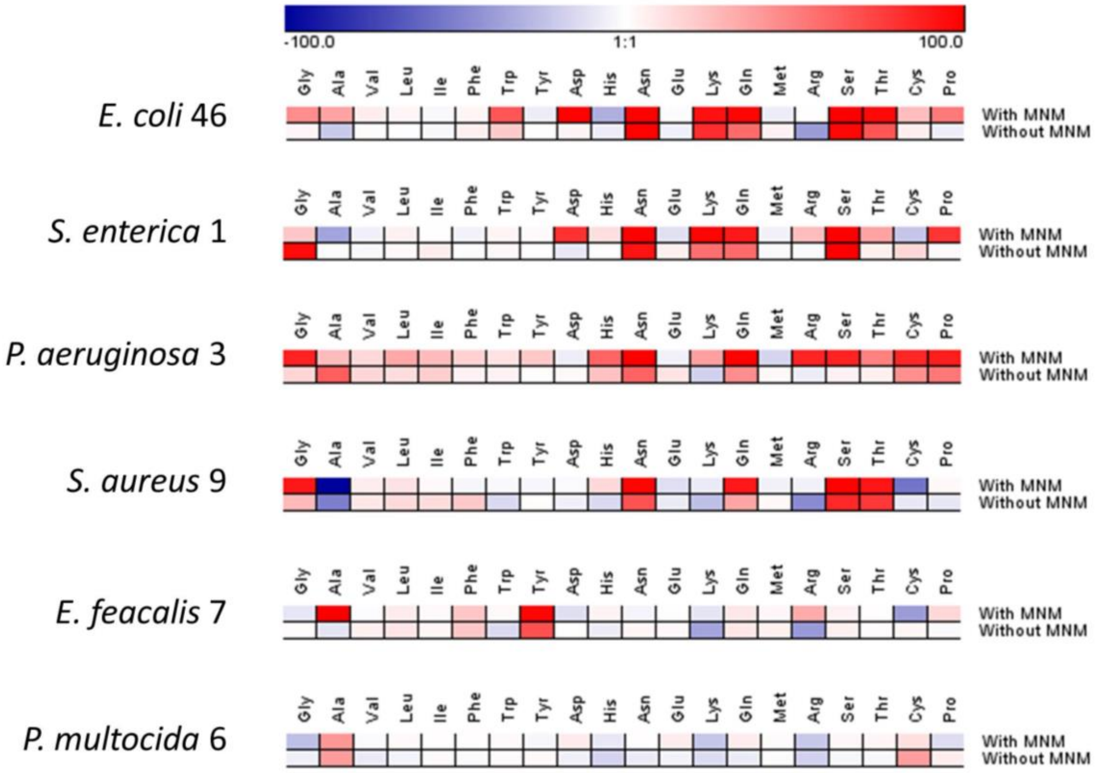

Supplementary Figure 4. The effect of MNM on the determination of AA consumption. Strains from six bacterial species were selected to evaluate the influence of MNM. Each strain was cultivated in 20 AAs medium with and without MNM, and then the consumption level of AA was determined after cultivation. The color gradients, which goes from white to red and white to blue, represent the level of positive consumption (0 to 100%) and negative consumption (0 to -100%) of AAs, respectively.

Supplementary Figure 5. Schematic diagram of the experimental procedure.

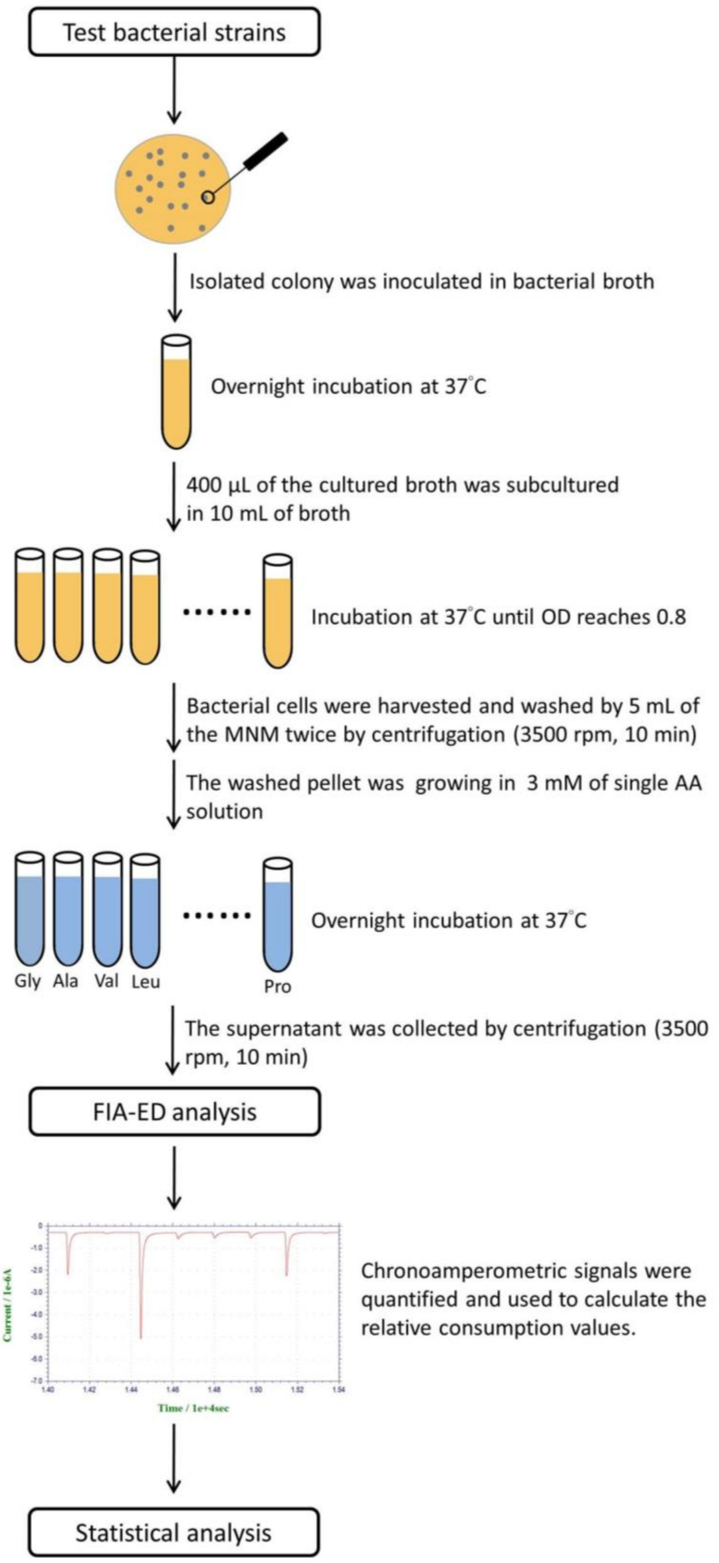

	Bacterial strains	Source	Note
No. 1	*Escherichia coli*	Pig, intestine	
No. 2	*Escherichia coli*	Pig, intestine	
No. 3	*Escherichia coli*	Pig, intestine	
No. 4	*Escherichia coli*	Pig, intestine	
No. 5	*Escherichia coli*	Pig, intestine	
No. 6	*Escherichia coli*	Pig, intestine	
No. 7	*Escherichia coli*	Pig, intestine	
No. 8	*Escherichia coli*	Pig, intestine	
No. 9	*Escherichia coli*	Pig, intestine	
No. 10	*Escherichia coli*	Pig, intestine	
No. 11	*Escherichia coli*	Chicken, uterine tube	
No. 12	*Escherichia coli*	Chicken, lung	
No. 13	*Escherichia coli*	Chicken, liver	
No. 14	*Escherichia coli*	Chicken, nasal mucus	
No. 15	*Escherichia coli*	Chicken, intestine	
No. 16	*Escherichia coli*	Chicken, intestine	
No. 17	*Escherichia coli*	Cat	
No. 18	*Escherichia coli*	Cat	
No. 19	*Escherichia coli*	Cat	
No. 20	*Escherichia coli*	Cat	
No. 21	*Escherichia coli*	Cat	
No. 22	*Escherichia coli*	Dog	
No. 23	*Escherichia coli*	Dog	
No. 24	*Escherichia coli*	Dog	
No. 25	*Escherichia coli*	Dog	
No. 26	*Escherichia coli*	Dog	
No. 27	*Escherichia coli*	Goose, joint	
No. 28	*Escherichia coli*	Goose, lung	
No. 29	*Escherichia coli*	Goose, bursa of Fabricius	
No. 30	*Escherichia coli*	Goose, liver	
No. 31	*Escherichia coli*	Goose, lung	
No. 32	*Escherichia coli*	Pigeon, lung	
No. 33	*Escherichia coli*	Turkey, lung	
No. 34	*Escherichia coli*	Chicken, intestine	
No. 35	*Escherichia coli*	Chicken, intestine	
No. 36	*Escherichia coli*	Chicken, intestine	
No. 37	*Escherichia coli*	Chicken, intestine	

No. 38	*Escherichia coli*	Chicken, intestine	
No. 39	*Escherichia coli*	Chicken, intestine	
No. 40	*Escherichia coli*	Chicken, air sac	
No. 41	*Escherichia coli*	Chicken, intestine	
No. 42	*Escherichia coli*	Chicken, heart	
No. 43	*Escherichia coli*	Chicken, lung	
No. 44	*Escherichia coli*	Chicken, liver	
No. 45	*Escherichia coli*	Chicken, lung	
No. 46	*Escherichia coli*		ATCC 25922
No. 47	*Escherichia coli*		ATCC 35150
No. 48	*Escherichia coli*		colistin resistant
No. 49	*Escherichia coli*		colistin resistant
No. 50	*Escherichia coli*		colistin resistant
No. 51	*Escherichia coli*		colistin resistant
No. 52	*Escherichia coli*		colistin resistant
No. 1	*Pasteurella multocida*	Pig, lung	Type D
No. 2	*Pasteurella multocida*	Pig, lung	Type D
No. 3	*Pasteurella multocida*	Pig, lung	Type D
No. 4	*Pasteurella multocida*	Pig, lung	Type D
No. 5	*Pasteurella multocida*	Pig, lung	Type D
No. 6	*Pasteurella multocida*	Pig, lung	Type D
No. 7	*Pasteurella multocida*	Pig, lung	Type D
No. 8	*Pasteurella multocida*	Pig, lung	Type D
No. 9	*Pasteurella multocida*	Pig, lung	Type D
No. 10	*Pasteurella multocida*	Pig, lung	Type D
No. 11	*Pasteurella multocida*	Pig, joint	Type D
No. 12	*Pasteurella multocida*	Pig, lung	Type D
No. 13	*Pasteurella multocida*	Pig, lung	Type D
No. 14	*Pasteurella multocida*	Pig, lung	Type A
No. 15	*Pasteurella multocida*	Pig, lung	Type A
No. 16	*Pasteurella multocida*	Pig, lung	Type A
No. 17	*Pasteurella multocida*	Pig, lung	Type A
No. 18	*Pasteurella multocida*	Pig, lung	Type A
No. 19	*Pasteurella multocida*	Pig, lung	Type A
No. 20	*Pasteurella multocida*	Pig, lung	Type A
No. 21	*Pasteurella multocida*	Pig, lung	Type A
No. 1	*Salmonella enterica*	Chicken, liver	
No. 2	*Salmonella enterica*	Chicken, liver	

No. 3	*Salmonella enterica*	Chicken, liver	
No. 4	*Salmonella enterica*	Chicken, liver	
No. 5	*Salmonella enterica*	Chicken, liver	
No. 6	*Salmonella enterica*	Chicken, liver	
No. 7	*Salmonella enterica*	Chicken, liver	
No. 8	*Salmonella enterica*	Chicken, liver	
No. 9	*Salmonella enterica*	Chicken, liver	
No. 10	*Salmonella enterica*	Chicken, liver	
No. 11	*Salmonella enterica*	Chicken, liver	
No. 12	*Salmonella enterica*	Chicken, liver	
No. 13	*Salmonella enterica*	Chicken, liver	
No. 14	*Salmonella enterica*	Chicken, liver	Schwarzengrund
No. 15	*Salmonella enterica*	Turkey, diet	Schwarzengrund
No. 16	*Salmonella enterica*	Turkey, feces	Albany
No. 17	*Salmonella enterica*	Turkey, diet	Schwarzengrund
No. 18	*Salmonella enterica*	Turkey, feces	Schwarzengrund
No. 19	*Salmonella enterica*	Goose, spleen	Typhimurium
No. 20	*Salmonella enterica*	Goose, pool	Albany
No. 21	*Salmonella enterica*	Pig, liver	Hadar
No. 22	*Salmonella enterica*	Pig, liver	Typhimurium
No. 23	*Salmonella enterica*	Pig, liver	Typhimurium
No. 24	*Salmonella enterica*	Pig, liver	Typhimurium
No. 25	*Salmonella enterica*	Pig, liver	Typhimurium
No. 26	*Salmonella enterica*	Duck, feces	Typhimurium
No. 27	*Salmonella enterica*	Sparrow, liver	Albany
No. 1	*Pseudomonas aeruginosa*	Dog, synovial fluid	
No. 2	*Pseudomonas aeruginosa*	Dog, urine	
No. 3	*Pseudomonas aeruginosa*	Dog, pus	
No. 4	*Pseudomonas aeruginosa*	Dog, pus	
No. 5	*Pseudomonas aeruginosa*	Dog, urine	
No. 6	*Pseudomonas aeruginosa*	Dog, bile	
No. 7	*Pseudomonas aeruginosa*	Dog, ear	
No. 8	*Pseudomonas aeruginosa*	Pig, liver	
No. 9	*Pseudomonas aeruginosa*	Pig, intestine	
No. 10	*Pseudomonas aeruginosa*	Pig, liver	
No. 11	*Pseudomonas aeruginosa*	Pig, kedney	
No. 12	*Pseudomonas aeruginosa*	Pig, lung	
No. 13	*Pseudomonas aeruginosa*	Goose, liver	

No. 14	*Pseudomonas aeruginosa*	Goose, liver
No. 15	*Pseudomonas aeruginosa*	Goose, brain
No. 16	*Pseudomonas aeruginosa*	Goose, liver
No. 17	*Pseudomonas aeruginosa*	Goose, lung
No. 18	*Pseudomonas aeruginosa*	Rabbit, pus
No. 19	*Pseudomonas aeruginosa*	Rabbit, pus
No. 20	*Pseudomonas aeruginosa*	Rabbit
No. 21	*Pseudomonas aeruginosa*	Sheep, breast
No. 22	*Pseudomonas aeruginosa*	Sheep, breast
No. 23	*Pseudomonas aeruginosa*	Chicken, air sac
No. 24	*Pseudomonas aeruginosa*	Chicken, lung
No. 25	*Pseudomonas aeruginosa*	Duck, egg
No. 26	*Pseudomonas aeruginosa*	Turtle, pus
No. 27	*Pseudomonas aeruginosa*	Plant
No. 28	*Pseudomonas aeruginosa*	Plant
No. 1	*Sataphylococcus aureus*	Chicken, joint
No. 2	*Sataphylococcus aureus*	Chicken, joint
No. 3	*Sataphylococcus aureus*	Chicken, liver
No. 4	*Sataphylococcus aureus*	Chicken, joint
No. 5	*Sataphylococcus aureus*	Chicken, liver
No. 6	*Sataphylococcus aureus*	Chicken, air sac
No. 7	*Sataphylococcus aureus*	Chicken, liver
No. 8	*Sataphylococcus aureus*	Chicken, lung
No. 9	*Sataphylococcus aureus*	Chicken, joint
No. 10	*Sataphylococcus aureus*	Chicken, joint
No. 11	*Sataphylococcus aureus*	Chicken, joint
No. 12	*Sataphylococcus aureus*	Chicken, liver
No. 13	*Sataphylococcus aureus*	Chicken, joint
No. 14	*Sataphylococcus aureus*	Chicken, liver
No. 15	*Sataphylococcus aureus*	Chicken, joint
No. 16	*Sataphylococcus aureus*	Chicken, air sac
No. 17	*Sataphylococcus aureus*	Chicken, joint
No. 18	*Sataphylococcus aureus*	Chicken, heart
No. 19	*Sataphylococcus aureus*	Chicken, joint
No. 20	*Sataphylococcus aureus*	Pig, lung
No. 21	*Sataphylococcus aureus*	Cattle, milk
No. 22	*Sataphylococcus aureus*	Cattle, milk
No. 23	*Sataphylococcus aureus*	Cattle, milk

No. 24	*Sataphylococcus aureus*	Cattle, milk	
No. 25	*Sataphylococcus aureus*	Cattle, milk	
No. 26	*Sataphylococcus aureus*		
No. 27	*Sataphylococcus aureus*	Chicken, muscle	MRSA
No. 28	*Sataphylococcus aureus*	Chicken, muscle	MRSA
No. 29	*Sataphylococcus aureus*	Chicken, muscle	MRSA
No. 30	*Sataphylococcus aureus*	Chicken, muscle	MRSA
No. 31	*Sataphylococcus aureus*	Chicken, muscle	MRSA
No. 32	*Sataphylococcus aureus*	Chicken, muscle	MRSA
No. 33	*Sataphylococcus aureus*	Chicken, muscle	MRSA
No. 34	*Sataphylococcus aureus*	Chicken, muscle	MRSA
No. 35	*Sataphylococcus aureus*	Chicken, muscle	MRSA
No. 36	*Sataphylococcus aureus*	Chicken, muscle	MRSA
No. 37	*Sataphylococcus aureus*	Human	
No. 38	*Sataphylococcus aureus*	Human	
No. 39	*Sataphylococcus aureus*	Human	
No. 40	*Sataphylococcus aureus*	Human	
No. 41	*Sataphylococcus aureus*	Human	
No. 42	*Sataphylococcus aureus*	Human	MRSA
No. 43	*Sataphylococcus aureus*	Human	MRSA
No. 44	*Sataphylococcus aureus*	Human	MRSA
No. 45	*Sataphylococcus aureus*	Human	MRSA
No. 1	*Enterococcus faecalis*	Duck, trachea	
No. 2	*Enterococcus faecalis*	Duck, trachea	
No. 3	*Enterococcus faecalis*	Duck, trachea	
No. 4	*Enterococcus faecalis*	Duck, lung	
No. 5	*Enterococcus faecalis*	Duck, lung	
No. 6	*Enterococcus faecalis*	Duck, lung	
No. 7	*Enterococcus faecalis*	Duck, trachea	
No. 8	*Enterococcus faecalis*	Chicken, feces	
No. 9	*Enterococcus faecalis*	Chicken, feces	
No. 10	*Enterococcus faecalis*	Chicken, feces	
No. 11	*Enterococcus faecalis*	Chicken, feces	
No. 12	*Enterococcus faecalis*	Goose, feces	
No. 13	*Enterococcus faecalis*	Goose, feces	
No. 14	*Enterococcus faecalis*	Goose, feces	
No. 15	*Enterococcus faecalis*	Goose, feces	
No. 16	*Enterococcus faecalis*	Bird, feces	

No. 17	*Enterococcus faecalis*	Bird, feces
No. 18	*Enterococcus faecalis*	Bird, feces
No. 19	*Enterococcus faecalis*	Bird, feces
No. 20	*Enterococcus faecalis*	Bird, feces
No. 21	*Enterococcus faecalis*	Bird, feces
No. 1	*Staphylococcus hyicus*	Pig, joint
No. 2	*Staphylococcus hyicus*	Pig, joint
No. 3	*Staphylococcus hyicus*	Pig, skin
No. 4	*Staphylococcus hyicus*	Pig, skin
No. 5	*Staphylococcus hyicus*	Pig, skin
No. 6	*Staphylococcus hyicus*	Pig, joint
No. 7	*Staphylococcus hyicus*	Pig
No. 8	*Staphylococcus hyicus*	Pig
No. 9	*Staphylococcus hyicus*	Pig
No. 10	*Staphylococcus hyicus*	Pig
No. 11	*Staphylococcus hyicus*	Pig
No. 12	*Staphylococcus hyicus*	Pig
No. 13	*Staphylococcus hyicus*	Pig
No. 14	*Staphylococcus hyicus*	Pig
No. 15	*Staphylococcus hyicus*	Pig
No. 16	*Staphylococcus hyicus*	Pig
No. 17	*Staphylococcus hyicus*	Pig
No. 18	*Staphylococcus hyicus*	Pig
No. 19	*Staphylococcus hyicus*	Pig

Supplementary Table 1. All clinical isolates and reference strains.

All animal isolates and all human isolates are provided from Dr. Hung-Chih Kuo’s laboratory and Dr. Chih-Ho Lai's laboratory, respectively.
